# Minimal important change values for the Oxford Knee Score and the Forgotten Joint Score at 1 year after total knee replacement

**DOI:** 10.1080/17453674.2018.1480739

**Published:** 2018-06-04

**Authors:** Lina H Ingelsrud, Ewa M Roos, Berend Terluin, Kirill Gromov, Henrik Husted, Anders Troelsen

**Affiliations:** a Department of Orthopaedic Surgery, Copenhagen University Hospital Hvidovre, Copenhagen, Denmark;;; b Department of Sports Science and Clinical Biomechanics, University of Southern Denmark, Odense, Denmark;;; c Department of General Practice and Elderly Care Medicine, VU University Medical Center, Amsterdam, Netherlands

## Abstract

Background and purpose — Interpreting changes in Oxford Knee Score (OKS) and Forgotten Joint Score (FJS) following total knee replacement (TKR) is challenged by the lack of methodologically rigorous methods to estimate minimal important change (MIC) values. We determined MIC values by predictive modeling for the OKS and FJS in patients undergoing primary TKR.

Patients and methods — We conducted a prospective cohort study in patients undergoing TKR between January 2015 and July 2016. OKS and FJS were completed preoperatively and at 1 year postoperatively, accompanied by a 7-point anchor question ranging from “better, an important improvement” to “worse, an important worsening.” MIC improvement values were defined with the predictive modeling approach based on logistic regression, with patients’ decisions on important improvement as dependent variable and change in OKS/FJS as independent variable. Furthermore, the MICs were adjusted for high proportions of improved patients.

Results — 333/496 (67.1%) patients with a median age of 69 years (61% female) had complete data for OKS, FJS, and anchor questions at 1 year postoperatively. 85% were importantly improved. Spearman’s correlations between the anchor and the change score were 0.56 for OKS, and 0.61 for FJS. Adjusted predictive MIC values (95% CI) for improvement were 8 (6–9) for OKS and 14 (10–18) for FJS.

Interpretation — The MIC value of 8 for OKS and 14 for FJS corresponds to minimal improvements that the average patient finds important and aids in our understanding of whether improvements after TKR are clinically relevant.

Patient-reported outcome measures (PROM) are increasingly advocated as primary outcome measures in clinical trials, as well as quality of care assessment in arthroplasty registries (FDA and HHS 2009, Rolfson et al. 2016). The Oxford Knee Score (OKS) and Forgotten Joint Score (FJS) are 2 such commonly used outcome measures developed for patients undergoing TKR (Giesinger et al. 2014, Harris et al. 2016, Thomsen et al. 2016). However, interpreting whether changes in OKS and FJS scores are clinically meaningful is challenging because statistically significant improvements are not necessarily clinically meaningful (King 2011).

The concept of minimal important change (MIC) is defined as the smallest change in a PROM considered important by a notional average patient (Terluin et al. 2015). MIC values for PROMs may differ depending on the patient population, intervention, follow-up time etc. It is therefore necessary to determine context-specific MIC threshold values for specific PROMs that may improve the translation of PROM scores into clinical relevance (King 2011).

No previous studies have estimated MIC values for the FJS. MIC values for OKS ranging from 7 to 9 at 6 months following a TKR, and 4.3 to 5 at 12 months follow-up have been suggested (Clement et al. 2014, Beard et al. 2015). However, the applicability of the reported MIC values depends on the definition of MIC and the methodological approach used to determine the MIC values. As different methodological approaches yield different MIC values (Terluin et al. 2015), additional studies are needed to further establish MIC thresholds for the OKS. We therefore determined MIC values for the OKS and FJS at 1 year after a TKR. These MIC values are intended for interpretation of within-group mean improvements and for use as responder thresholds when interpreting whether improvements differ between intervention groups, including the surveillance of treatment outcome in registries and clinical studies as a supplement to implant survivorship.

## Patients and methods

### Study design and setting

The study is a prospective observational cohort study using data from 1 Danish hospital’s local arthroplasty registry. From March 2013, all patients scheduled for joint replacement at the hospital were asked to complete an electronic questionnaire at their preoperative visit at the hospital. At 1 year postoperatively, patients received an email with a link to an electronic follow-up questionnaire. If the questionnaire was not completed after 2 reminder emails with a 2-week interval, a paper version of the questionnaire was sent by postal mail.

### Participants

Data from all patients with knee replacement surgery performed between January 1, 2015 and July 31, 2016 were extracted from the registry. Patients who had revision surgery and unicompartmental arthroplasty were excluded. To avoid multiple observations on patients having had surgery performed on both knees within the data extraction period, or simultaneous bilateral surgery, we randomly selected 1 of the observations to be included in the analyses.

### Questionnaires

Preoperative and 1-year postoperative questionnaires that were extracted from the registry included the OKS, FJS, and an anchor question. The OKS and FJS were developed to evaluate patient-relevant outcomes after TKR. Previous studies have established adequate validity, reliability, and responsiveness characteristics for the OKS and FJS in the TKR population (Behrend et al. 2012, Harris et al. 2016, Thomsen et al. 2016, Hamilton et al. 2017). The OKS includes 12 items about knee pain and function that are summed to a total score of 0–48 (worst–best) (Murray et al. 2007). The FJS includes 12 items about the patient’s knee awareness in different daily life activities. The original version that was developed in 2012 (Behrend et al. 2012), and cross-culturally adapted into Danish, asked patients “Are you aware of your artificial knee…” (Thomsen et al. 2016). The FJS was later shown to be responsive in measuring change in knee awareness from before to after a TKR (Thienpont et al. 2016, Hamilton et al. 2017). Therefore, to enable measurement of change from before to after surgery in our study, the question in the preoperative form was adapted to “Are you aware of your knee…”, in accordance with the version used in the British population (Hamilton et al. 2017). A total score of 0 to 100 is calculated, with higher scores reflecting higher ability to forget the knee joint (Behrend et al. 2012). Missing items in OKS and FJS were handled in accordance with each respective user-guide (Murray et al. 2007, Behrend et al. 2012).

Furthermore, at 1 year postoperatively, the patients’ experienced degree and importance of change were obtained by asking the anchor question: “How are your knee problems now compared to prior to your operation?” Patients responded on a 7-point scale ranging from “better, an important improvement” to “worse, an important deterioration” (Jaeschke et al. 1989). Patients were classified as being importantly improved when answering “better, an important improvement” or “somewhat better, but enough to be an important improvement” (Table 1).

**Table 1.** Response options to minimal important change anchor question and classification into importantly improved or not

Classification of importance/Response options
**Importantly improved**
n Better, an important improvement
n Somewhat better, but enough to be an important improvement
**Not importantly improved**
n Very small change, not enough to be an important improvement
n About the same
n Very small change, not enough to be an important deterioration
n Somewhat worse, but enough to be an important deterioration
n Worse, an important deterioration

## Statistics

Patient demographics were presented as median (interquartile range (IQR)) for non-normally distributed continuous variables and number (proportion) for categorical variables. OKS and FJS change score distributions across anchor response categories were investigated with boxplots. The association between the OKS and FJS change score and anchor responses were investigated with Spearman’s correlation. R version 3.4.0 (https://www.r-project.org/) was used for all analyses.

## Anchor-based MIC approach

MIC values were estimated using anchor-based approaches that involve anchoring of the OKS and FJS change scores to the anchor question responses. Several anchor-based methods are advocated and there is no consensus on which method provides the best estimate of MIC values (King 2011). Due to its methodological advantages, our primary method for estimating MIC values is the newly described predictive modeling method (MIC_pred_) (Terluin et al. 2015). This method is more precise than the commonly used receiver operating characteristics (ROC) method and less dependent on the correlation between the PROM score and the anchor responses (Terluin et al. 2015). Furthermore, it enables adjustment for the bias that incurs when the proportion of improved patients is smaller or larger than 50%. This adjusted MIC_pred_ has been shown to equal the mean of the latent individual MICs. Hence, the adjusted MIC_pred_ represents the amount of change that the notional average patient considers to be minimally important. The predictive modeling method is based on a logistic regression, using the dichotomized anchor response (importantly improved or not) as dependent variable and the PROM change score as independent variable. The change in PROM that corresponds to a Likelihood Ratio of 1 is estimated as the MIC value. With a likelihood ratio of 1, the posttest odds of being importantly improved are the same as the pretest odds of improvement. The adjustment for the large proportion of improved patients was performed with the equation

$${\text{MIC}}_{\text{adjusted}}={\text{ MIC}}_{\text{pred}}–\;\left(0.0\text{9}0\;+\;0.\text{1}0\text{3 }\times \text{ Cor}\right)\;\times {\text{ SD}}_{\text{change}}\times log-odds\left(imp\right)$$

*Cor* is the point biserial correlation between the PROM change score and the anchor, *SD_change_* is the SD of the change score, and *log-odds(imp)* is the natural logarithm of (proportion improved/[1 – proportion improved]). Bootstrap replications (n = 1,000) were used to determine 95% confidence intervals (CI) for adjusted MIC_pred_ (Terluin et al. 2017).

To enable comparison with other commonly described methods, we also estimated MIC values with the mean change (MIC_MeanChange_) and ROC (MIC_ROC_) methods. With the mean change method the MIC value corresponds to the mean change in PROM in the subgroup of patients responding “somewhat better, enough to be importantly improved” (Jaeschke et al. 1989). We calculated 95% CI for the ­*MIC_MeanChange_* as *Mean_change_ ± 1.96 (SD_change_/(*√*n))*, with *n* and *SD_change_* corresponding to the subgroup “somewhat better.” With the ROC method, the MIC value is the change in PROM score that with the least degree of misclassification, according to the Youden criterion, discriminates patients from being importantly improved or not. Bootstrap replications (n = 1,000) were used to determine 95% CI for MIC_ROC_ (Terwee et al. 2010).

## Baseline dependency

To investigate whether preoperative severity impacted on MIC_pred_ values, an interaction term between the preoperative PROM score and change in PROM score was included in each respective logistic regression model (Terluin et al. 2015). Effect modification of MIC_pred_ was considered present if interaction terms had p-values <0.05.

## Ethics, funding, and potential conflicts of interest

The local arthroplasty registry was approved by the national data protection agency (Journal number HVH-2012-048). In Denmark, approval from the ethical committee is not required for register-based studies involving only questionnaire data. The study was conducted in accordance with the WMA Declaration of Helsinki. The study was fully funded by the orthopedic department at the hospital. The authors declare that there are no potential conflicts of interest in relation to this study.

## Results

### Participants

**Figure 1.**
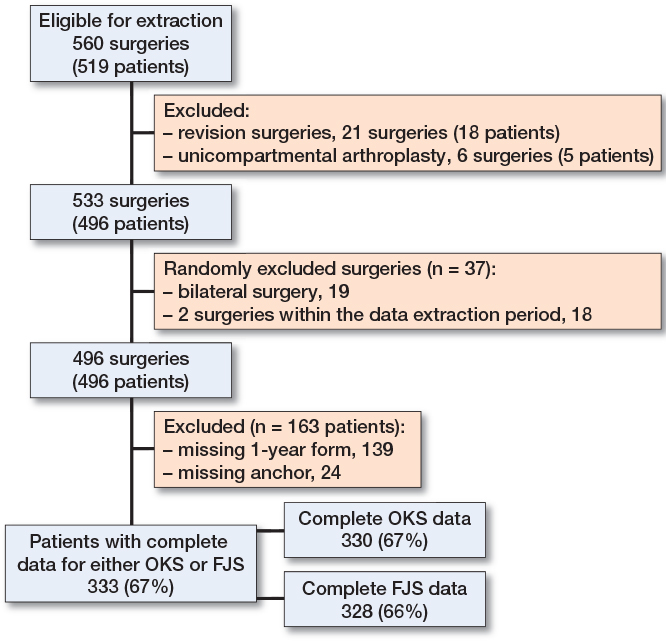
Flow chart.

After excluding patients who had undergone revision surgery or unicompartmental arthroplasty, 496 unique patients were registered with a primary TKA, of which 139 were excluded because they had not answered the 1-year follow-up form. Complete data for anchor questions and for either the OKS or FJS were available for 333/496 (67%) patients (Figure 1).

### Preoperative patient characteristics

Patients with complete data had a median (IQR) age of 69 (61–73) years and 61% were female. These patients differed from the patients with incomplete data, with 12% fewer females (p = 0.01) and a 2-point higher median preoperative OKS (p = 0.01). Other preoperative characteristics were comparable (Table 2).

**Table 2.** Patient preoperative demographics. Values are median (interquartile range), unless otherwise stated

	Patients with	Patients with	
	complete data	incomplete data	
	n = 333	n = 163	p-value**^**a**^**
Age	69 (61–73)	68 (61–75)	0.7
Female, n (%)	203 (61)	119 (73)	0.01
Body mass index	29 (26–33)	30 (26–33)	0.8
Oxford Knee Score	23 (17–27)	21 (15–25)	0.01
Forgotten Joint Score	14 (6–27)	14 (7–23)	0.6

**^a^**Wilcoxon Signed Rank test for continuous variables and chi-square test for dichotomous variables.

### Descriptive data

**Figure 2.**
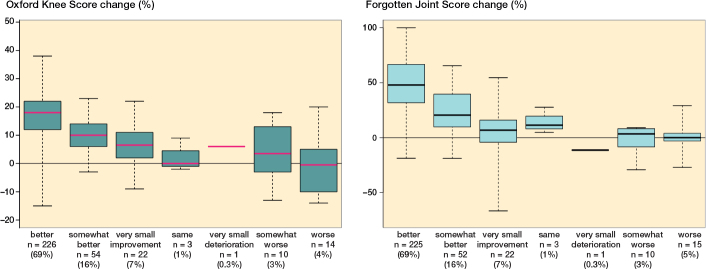
OKS and FJS change scores by anchor questions response categories ranging from “better, an important improvement” to “worse, an important deterioration.” Horizontal bars present the median, the box the interquartile range, and the whiskers the maximum and minimum scores.

The overall percentage of patients reporting important improvements was 85%, while 8% reported being either unchanged, or perceiving too small improvement or deterioration to be of importance, and the final 8% reported being importantly deteriorated. OKS and FJS change scores for each of the anchor response categories are presented in Figure 2.

### MIC improvement values

The correlations between the anchor question and the change in OKS and FJS were 0.56 and 0.61, respectively.

MIC_pred_ improvement values adjusted for the large proportions of improved patients were 8 (CI 6–9) for OKS and 14 (CI 10–18) for FJS. Unadjusted MIC values determined with the mean change method and the ROC method were higher with wider 95% CI than the adjusted MIC_pred_ values (Table 3).

**Table 3.** MIC improvement values determined with the predictive modeling approach adjusted for the proportions of improved patients, the mean change method and the ROC method

	Predictive	Mean change			
	modeling approach**^**a**^**	method	ROC method		
	MIC_pred_ (CI**^**b**^**)	MIC_MeanChange_ (CI**^**c**^**)	MIC_ROC_ (CI**^**b**^**)	Sensitivity	Specificity
Oxford Knee Score	8 (6–9)	10 (8–1)	9 (6–15)	0.83	0.74
Forgotten Joint Score	14 (10–18)	23 (17–28)	17(11–29)	0.85	0.88

**^a^**adjusted for the proportions improved.

**^b^**95% confidence intervals (CI) calculated using 1,000 bootstrap replications, reported as 0.025–0.975 quantiles.

**^c^**95% confidence intervals (CI) calculated as *Mean_change_ ± 1.96 (SD_change_/(√n))*, where *n* and *SD_change_* correspond

## Baseline dependency

Interaction terms between preoperative and change in OKS and FJS scores were statistically not significant (p = 0.1 and p = 0.9, respectively), suggesting no baseline dependency of MIC_pred_ values.

to the subgroup “somewhat better.”

## Discussion

### Summary of findings

In this prospective single-center study we propose estimates for meaningful improvement in OKS and FJS at one year after primary TKR. The MICpred values of 8 for the OKS and 14 for the FJS reflect the smallest improvement needed to be considered important by a notional average patient one year after a TKR. The majority of patients (85%) experienced important improvements in their knee problems, while 8% considered themselves to be unchanged and 8% to have importantly deteriorated after TKR.

### Relation to previous studies

No previous studies have determined MIC values for the FJS with which we can compare our findings. Our MIC value for the OKS lies in the range of those proposed by Beard et al. (2015). They used data on 94,015 patients from the NHS PROMS data set, and found that 6 months after surgery changes larger than 6.5 calculated with the ROC method and 9.2 points calculated with the mean change method were considered clinically meaningful. The similarity in MIC estimates between our studies suggests that the thresholds for important improvements may not vary much between 6 and 12 months after TKR. Conversely, OKS MIC values proposed by Clement et al. (2014) were smaller, ranging from 4.3 to 5 at 12 months after a TKR. Their anchor question, a 5-point Likert scale of satisfaction with functional improvement and pain relief, and statistical approach, a simple linear regression, differed from our study, which may explain the discrepancy in MIC estimates (Clement et al. 2014). Although our proposed MIC values are in the same range as those from the study by Beard et al. (2015), methodological differences may explain the variation in MIC estimates that have been found (Terwee et al. 2010).

### MIC estimations vary with methodology

In accordance with a previous study, we found that MIC values differ with methodology used (Ingelsrud et al. 2018). For both the OKS and the FJS we found the largest MIC values with the mean change method, and after adjusting for the large proportion of improved patients the smallest MIC values were found with the predictive modeling method.

Although the mean change method is appealing because it is intuitive and easily calculated, it is criticized because only data from a subgroup of the population sample are used (Terwee et al. 2010, King 2011). Furthermore, MIC_MeanChange_ values are not considered appropriate as responder criteria because, assuming normal distribution of scores, only half of the patients in the subgroup used to calculate the threshold value would be correctly classified as being importantly improved (McLeod et al. 2011).

Conversely, with the ROC method, all data points are used in the MIC estimation, but simulation studies have shown it to be less precise and more susceptible to errors than the predictive modeling method (Terluin et al. 2015). As an example, the optimal ROC cut-off of 8.5 in our study was associated with smaller degrees of misclassification (specificity: 0.74 and sensitivity: 0.83) than the cut-off of 6.5 found by Beard et al. (2015) (specificity: 0.64 and sensitivity: 0.65). We consider the discrepancy between these MIC_ROC_ values to result from the impreciseness of the ROC method, probably due to random fluctuations in the samples. The ROC method’s impreciseness is also revealed from the wider CI in our study as compared with the CI for the MIC_pred_ (Table 3) (Terluin et al. 2015).

Finally, the ROC method has been shown to yield the same result as the predictive modeling method when the change scores under study are perfectly normally distributed (Terluin et al. 2015). However, MIC_ROC_ values cannot be adjusted for the biased overestimation that results from proportions of improved patients being larger than 50%. The predictive modeling method is therefore preferred due to its strengths that include higher precision than the ROC method, and the ability to adjust for the overestimation resulting from proportions of improved patients being larger than 50% (Terluin et al. 2017).

### Limitations of our study

Limitations of our study include the risk of selection bias since almost 30% of the patients did not return their 1-year follow-up questionnaires. The non-responders were more often female and had a 2-point lower median OKS score (see Table 2). However, these differences are considered small, and as the responders with complete data are otherwise comparable to non-responders with regards to age, BMI, and preoperative knee awareness, we do not expect that our MIC values would differ had we had a higher response rate. Additionally, patients in our cohort were comparable to patients included in the Danish Knee Arthroplasty Registry. The mean age reported by the national registry for patients undergoing a primary TKR has been 67 to 69 years and the cumulated proportion of females has been 61% since 1997, which supports the representativeness of our cohort (Odgaard et al. 2016). Furthermore, possible confounding factors of the MIC values could be mental and medical comorbidities, socioeconomic characteristics, and radiographic osteoarthritis severity and pattern. However, since the patients in our cohort include diversity of these characteristics, we consider our results to be generalizable to other cohorts and registry settings where the sample diversity is assumed similar.

The risk of recall bias has previously been pointed out as a limitation when using anchor questions to estimate MIC values. Recall bias is considered to be present when the anchor responses are more highly correlated to the PROM follow-up score than the change score (Guyatt et al. 2002, King 2011). However, Terluin et al. (2017) in a simulation study showed that after adjusting for the proportions of improved patients exceeding 50%, the bias introduced by increasing the dependency on the follow-up score was very small (Terluin et al. 2017). We therefore do not consider recall bias a limitation of our MICpred estimates. Another limitation of the anchor-based approach is the bias caused by response shift. Response shift implies that patients’ own judgments of their health state changes throughout the follow-up period, resulting in paradoxical responses to the anchor questions, compared to the changes seen in the PROM. The effects of response shift on MIC estimations and how to handle it are, however, not clear (Schwartz et al. 2017).

A further important acknowledgment is that the MIC for improvement cannot serve to estimate that of deterioration in knee problems (Crosby et al. 2003). Even though 7% of the patients considered themselves to be importantly deteriorated after surgery, the absolute number of deteriorated patients was too low to enable the calculation of MIC values for deterioration.

Lastly, while the FJS was originally intended to evaluate the postoperative cross-sectional outcome of joint replacement surgery, subsequent studies have reported high responsiveness to change from before to after surgery when using an adapted version of the questionnaire (Thienpont et al. 2016, Hamilton et al. 2017). Although the validity and reliability characteristics of the Danish version used in our study were determined only in patients at 1 to 4 years postoperatively, we consider the changes made to the questionnaire to enable measurement of the preoperative knee awareness to be minor and that a new validation study of the Danish version does not seem needed since the changes are in line with other language versions of the FJS.

### Implications of findings

Terluin et al. (2017) demonstrated that the adjusted MIC_pred_ represents the mean of the latent individual MICs in a sample. Thus, the presented adjusted MIC_pred_ values can be used to interpret mean change improvements within one group of patients at 1 year after a TKR. In national registries and longitudinal cohort studies, improvements exceeding 8 on the OKS and 14.0 on the FJS reflect improvements that are considered important by a notional average patient. To enable comparison between groups in randomized clinical trials or other comparative study types, the adjusted MIC_pred_ values may be used as thresholds for treatment response. These thresholds can be used in responder analyses to calculate the numbers and proportions of responders in each treatment arm (McLeod et al. 2011). The MIC values are not applicable for comparing mean change improvements between groups, or to be used in corresponding sample size estimations. Such minimal important difference (MID) values require careful consideration depending on the specific context under study and how to decide on MID values for a specific study is much discussed (Cook et al. 2015). We do not consider that MID values can be derived from longitudinal data from only 1 cohort of patients.

When evaluating individual patients’ improvement in the clinic, the adjusted MIC_pred_ values should not be considered absolute thresholds, but may be used in shared decision-making as references to what a notional average patient finds important (King 2011).

The presented MIC values are considered context-specific and should not be transferred to other patient populations, time-points, or interventions. However, the presented MIC values are considered applicable to other TKR cohorts with comparable demographic characteristics as in our cohort. Finally, the MIC represents the smallest improvement that is needed to be considered important by patients, but it does not necessarily represent the best possible outcome or the full potential of the treatment. Other determinants of outcome may therefore also be relevant to evaluate in the comparison of different interventions.

## Conclusion

The MIC for improvement values of 8 for the OKS and 14 for the FJS can be used to interpret longitudinal within-group score changes, or as responder criteria when comparing improvements between 2 groups at 1 year after TKR. In addition to improving the interpretation of results from research studies, the MIC values may also aid in monitoring quality of treatment through national registries.

Design of the study: LHI, AT, ER. Analyses: LHI. Interpretation of results: LHI, ER, BT, KG, HH, AT. Manuscript preparation: LHI, AT. Manuscript review and final acceptance of manuscript: LHI, ER, BT, KG, HH, AT.

The authors would like to thank the staff at the orthopedic department for managing the local database at a daily basis. They also thank statisticians Thomas Kallemose and Håkon Sandholdt for statistical assistance.

*Acta* thanks Madeleine King and Annette W-Dahl for help with peer review of this study.
